# Chronic diseases and comorbidities in adults with and without intellectual disabilities: comparative cross-sectional study in Dutch general practice

**DOI:** 10.1093/fampra/cmac042

**Published:** 2022-05-17

**Authors:** Milou van den Bemd, Bianca W M Schalk, Erik W M A Bischoff, Maarten Cuypers, Geraline L Leusink

**Affiliations:** Radboud Institute for Health Sciences, Department of Primary and Community Care, Radboud University Medical Center, Nijmegen, The Netherlands; Radboud Institute for Health Sciences, Department of Primary and Community Care, Radboud University Medical Center, Nijmegen, The Netherlands; Radboud Institute for Health Sciences, Department of Primary and Community Care, Radboud University Medical Center, Nijmegen, The Netherlands; Radboud Institute for Health Sciences, Department of Primary and Community Care, Radboud University Medical Center, Nijmegen, The Netherlands; Radboud Institute for Health Sciences, Department of Primary and Community Care, Radboud University Medical Center, Nijmegen, The Netherlands

**Keywords:** chronic disease, comorbidity, general practice, intellectual disability, population health, prevalence

## Abstract

**Background:**

Chronic disease and comorbidity patterns in people with intellectual disabilities (ID) are more complex than in the general population. However, incomplete understanding of these differences limits care providers in addressing them.

**Objective:**

To compare chronic disease and comorbidity patterns in chronically ill patients with and without ID in Dutch general practice.

**Methods:**

In this population-based study, a multi-regional primary care database of 2018 was combined with national population data to improve identification of adults with ID. Prevalence was calculated using Poisson regression to estimate prevalence ratios and 95% confidence intervals for the highest-impact chronic diseases (ischemic heart disease (IHD), cerebrovascular disease (CVD), diabetes mellitus (DM), and chronic obstructive pulmonary disease (COPD)) and comorbidities.

**Results:**

Information from 18,114 people with ID and 1,093,995 people without ID was available. When considering age and sex, CVD (PR = 1.1), DM (PR = 1.6), and COPD (PR = 1.5) times more prevalent in people with than without ID. At younger age, people with ID more often had a chronic disease and multiple comorbidities. Males with ID most often had a chronic disease and multiple comorbidities. Comorbidities of circulatory nature were most common.

**Conclusions:**

This study identified a younger onset of chronic illness and a higher prevalence of multiple comorbidities among people with ID in general practice than those without ID. This underlines the complexity of people with ID and chronic diseases in general practice. As this study confirmed the earlier onset of chronic diseases and comorbidities, it is recommended to acknowledge these age differences when following chronic disease guidelines.

**Key messages**

- Disease burden is higher in those with vs without intellectual disabilities (ID).
- Different age and sex patterns impact chronic disease prevalence in ID patients.
- At younger age, people with ID more often have a diagnosis of a chronic disease.
- People with ID more often have multiple comorbidities, and at earlier age.
- Males with ID have the highest burden of chronic diseases and comorbidities.

## Background

Intellectual disabilities (ID) are characterized by substantial limitations in adaptive behaviour and intellectual functioning, that are expressed in lower conceptual, social, and practical skills when compared with people without ID.^1^ Approximately 1% of the global population complies to the formal definition of ID, but under-recognition and under-registration of ID could imply a higher percentage in general practice.^2^ As people with ID may experience difficulties in understanding and communicating (symptoms of) diseases, it can be more challenging to diagnose and timely treat conditions, resulting in more avoidable hospitalizations and premature deaths as compared to people without ID.^3-5^ Additionally, multimorbidity is highly prevalent, and frailty occurs 15 years earlier in people with ID.^3, 6^

Epidemiological patterns thus substantially differ in people with versus without ID. However, a full understanding of this complexity in people with ID is hampered by incomplete and insufficient literature on several crucial aspects. The different age and sex distribution of people with ID compared with the general population, meaning their life expectancy is lower and males more often have ID than females,^2, 5^ should be considered when studying chronic disease patterns.^7^ As current literature fails to do so, it is unclear whether the highest-impact chronic diseases, i.e. ischaemic heart disease (IHD), cerebrovascular disease (CVD), diabetes mellitus (DM), and chronic obstructive pulmonary disease (COPD), are more prevalent in people with or without ID.^8, 9^ Furthermore, information on comorbidity patterns in people with ID, meaning occurrence and characteristics of additional conditions alongside a chronic disease ,^10^ is largely lacking. Although comorbidities are highly prevalent among people with ID^11, 12^ and complicate the provision of optimal healthcare, existing studies solely focus on smaller ID populations or do not consider comorbidity characteristics .^12-20^

Despite these increased needs for healthcare, community-dwelling people with ID rely on the non-ID-oriented setting of regular primary care for chronic disease detection and management.^21, 22^ An accurate insight in chronic diseases in people with ID is thus necessary to generate awareness among primary care providers on the need for early detection and adequately treating chronic diseases and concomitant comorbidities. This study aims to examine (i) differences in prevalence of IHD, CVD, DM, and COPD between people with and without ID and (ii) occurrences and characteristics of comorbidities in chronically ill people with ID and a chronic disease compared with those without ID in Dutch primary care.

## Methods

### Data sources and study population

In this cross-sectional population-based retrospective study, we selected patients registered within Nivel Primary Care Database (NPCD). Data collection took place in 2021. This database is representative of Dutch general practice by routinely collecting medical information from over a million patients registered with 420 general practices .^23, 24^ Adults registered in 2018 were selected. People were identified as having ID if their medical record contained the only code available within International Classification of Primary Care (ICPC) for ID, P85 (Mental retardation).^25^ In order to improve identification of people with ID, we retrieved information on use of long-term care and supportive services from databases at Statistics Netherlands for all individuals in NPCD. If presence of an ID was noted in any of the linked databases from Statistics Netherlands, individuals were also included in the ID-group. This method is elaborated upon elsewhere .^2^

### Ethics

All data were pseudonymized and accessible only in Statistics Netherlands’ secured research environment. This study complied with the governance orders of Nivel (NZR-00320.002) and Statistics Netherlands. Because this study concerns retrospective research with non-traceable information, Radboud University Medical Center’s Ethics Committee has waived the need for formal ethical assessment (2017-3921). Prior to analyses, the research aims, hypotheses, methods, and analysis plan were preregistered (https://osf.io/kwv68/). The STROBE guidelines for reporting observational data were followed.^26^

### Operationalizations

Chronic diseases and comorbidities were encoded using ICPC-2.^25^ Individuals were defined as having a chronic disease when IHD, CVD, DM, and/or COPD were present in 2018 in their medical file (Supplementary Table S1). Comorbidities were defined based on a previously developed algorithm to construct illness episodes in NPCD,^24^ in which 109 chronic conditions were identified. ID was excluded (ICPC code P85), as it already served as selector variable, leaving 108 comorbidities (Supplementary Table S2). Comorbidity occurrence was presented as percentage of people that have 2 or more comorbidities next to their chronic disease.

### Statistical analysis

Descriptive statistics of the study groups were presented as frequencies with percentages or means with standard deviations. Chronic disease prevalence was compared between people with and without ID using Poisson regression analysis, estimating prevalence ratios (PR) and 95% confidence intervals (CIs), both unadjusted and age and sex adjusted. To acknowledge the large sample size, *P*-values below 0.005 were considered statistically significant.^27^ The percentage of people with and without ID having a diagnosis of chronic disease was presented in percentages and shown for males and females, and in 5-year age groups. The amount of people with 2 or more comorbidities was calculated in percentages for people with and without ID for males and females, and in 5-year age groups. All analyses were conducted using SPSS (version 25.0).

## Results

### Demographics

**Fig. 1.**
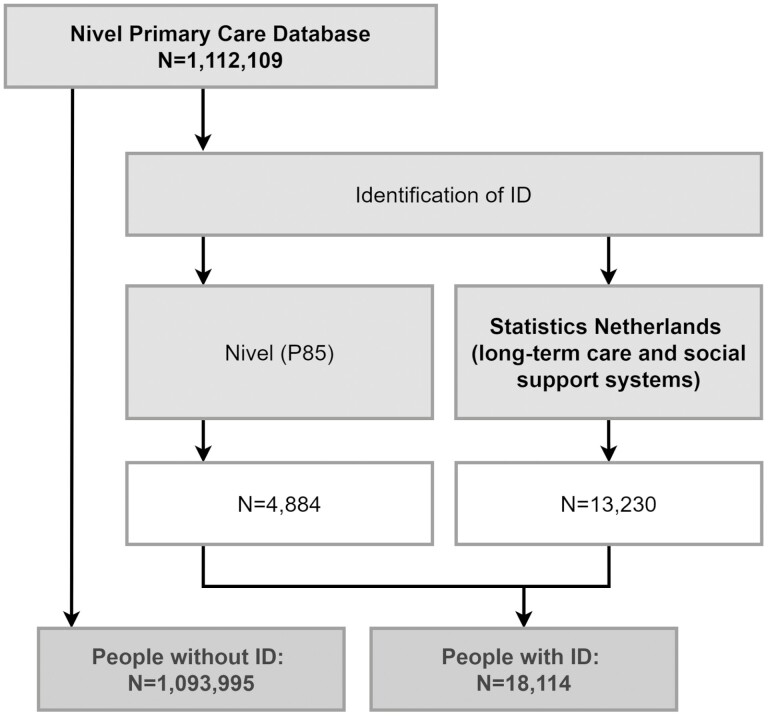
Identification of people with and without intellectual disabilities in final study groups.

The study groups consisted of 18,114 people with ID and 1,093,995 people without ID (Fig. 1). The percentage of males with ID (57.1%) was greater than the percentage of males without ID (48.8%) in their respective groups (Table 1). The average age of people with ID was lower than that of people without ID: 39.0 (SD: 15.9) versus 49.7 years (SD: 18.5), respectively. The majority of people with ID (71.1%) were younger than 50, with the largest group being 18–29 years (38.4%). Most of the people without ID were 50 years or older (50.4%); those aged 50–69 years were the largest group (33.9%). Of those with ID, 14.9% (*n* = 2,653) were diagnosed with at least one chronic disease; for those without ID, this was 16.9% (*n* = 184,681).

**Table 1.** Descriptive statistics of people with and without intellectual disabilities.

	People with ID *N* = 18,114	People without ID *N* = 1,093,995
Sex, *N* (%)		
Males	10,336 (57.1)	534,078 (48.8)
Females	7,778 (42.9)	559,917 (51.2)
Age, N (%)		
Mean age (SD)	39.0 (15.9)	49.7 (18.5)
Age groups, N (%)		
18–49 years	12,988 (71.1)	542,620 (49.6)
18–34 years	8,911 (49.2)	276,970 (25.3)
35–49 years	4,077 (22.5)	265,650 (24.3)
50 years or older	5,126 (28.3)	551,375 (50.4)
50–69 years	4,451 (24.6)	370,442 (33.9)
70 years or older	675 (3.7)	180,933 (16.5)
People with at least one chronic disease, *N* (%)	2,653 (14.9)	184,681 (16.9)

### Chronic disease prevalence

**Fig. 2.**
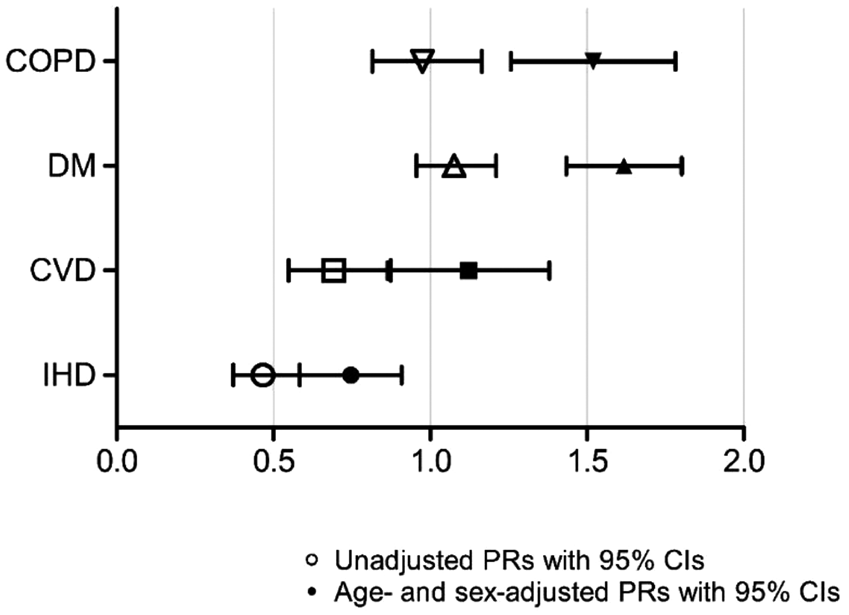
Chronic disease prevalence in unadjusted and sex- and age-adjusted prevalence ratios (PRs) in people with versus without intellectual disabilities.

Unadjusted for age and sex, IHD (PR = 0.47, CI = 0.43 to 0.51) and CVD (PR = 0.69, 0.63 to 0.76) were less common in people with than without ID (Fig. 2). People with ID more often had DM (PR = 1.08, CI = 1.03 to 1.13). There was no difference in COPD prevalence between those with and without ID (PR = 0.98, CI = 0.91 to 1.05). When adjusted for age and sex, different patterns emerged: prevalence rates all increased towards higher prevalence for people with ID. Unadjusted PRs ranged from 0.47 (IHD) to 1.08 (DM), while adjusted PRs ranged from 0.74 (IHD) to 1.62 (DM). In greater detail, CVD prevalence increased to PR = 1.12 (CI = 1.02 to 1.23), DM prevalence increased from PR = 1.08 to PR = 1.62 (CI = 1.54 to 1.69), and COPD prevalence became statistically significant (PR = 1.52, CI = 1.42 to 1.63).

**Fig. 3.**
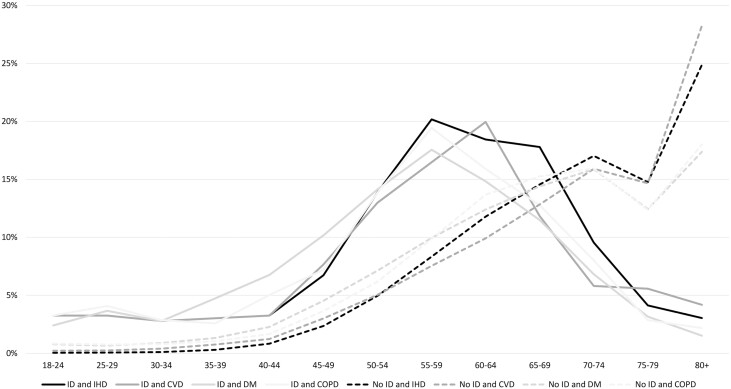
Percentage of people with and without intellectual disabilities that have a diagnosis of a chronic disease in 5-year age groups in 2018.

The onset of any of the observed chronic illnesses was at younger age for people with ID when compared with people without ID (Figure 3). In the age groups 18–24 years, prevalence was 3 to 5 times higher for people with ID than for people without ID. At ages 55–59 years, this difference was highest: to illustrate, IHD occurred in 20.2% of those with ID vs 8.3% of those without ID. Highest prevalence of any chronic illness among people with ID appeared in age groups below 70 years of age, while highest prevalence rates among people without ID are found among those aged 80 years or older, the percentage having CVD was highest among people without ID (28.3%), compared to 4.2% of those with ID (Supplementary Table S3).

Males with ID more often had a diagnosis of IHD, CVD, DM, or COPD than males without ID (not shown in figure; see Supplementary Table S3). For females with ID this pattern was reversed: females with ID less often had a diagnosis of a chronic disease than females without ID.

### Comorbidities

**Fig. 4.**
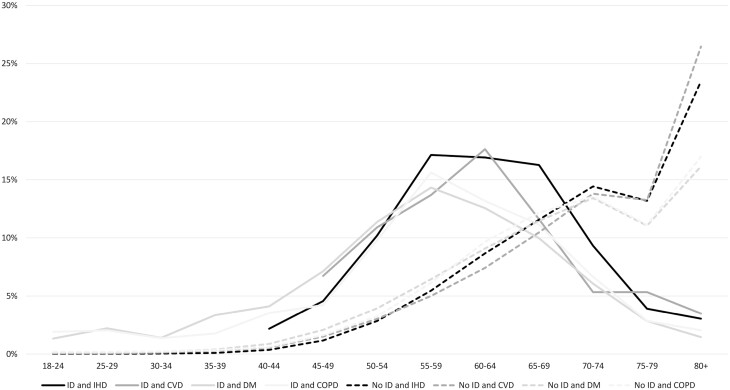
Percentage of people with and without intellectual disabilities having 2 or more comorbidities in 5-year age groups in 2018.

People with ID more often had comorbidities, at younger ages, and more often multiple comorbidities. In people with DM and COPD multiple comorbidities were more common in those with than without ID: 1.7% of 18–24 year olds with ID and DM had 2 or more comorbidities compared with 0.1% of those without ID. In COPD, 2.5% of 18–24 year olds with ID had 2 or more comorbidities compared to 0.3% of people without ID (Figure 4). In people with ID, the occurrence of 2 or more comorbidities was higher before age 65, while for people without ID it was highest after age 65. The highest occurrence of 2 or more comorbidities in people with ID with DM or COPD occurred at ages 55–59 years. 17.6% of people with ID and CVD aged 60–64 years had 2 or more comorbidities. For people with ID aged 70 years or older, the percentage with 2 or more comorbidities decreased, while it increased for those without ID. Of the 80-year old people with ID and CVD, 3.5% had 2 or more comorbidities, compared to 26.5% of those without ID (Supplementary Table S4).

Males with ID more often had 2 or more comorbidities next to their chronic disease of IHD, CVD, DM, or COPD compared with those without ID (not shown in figure; see Supplementary Table S4). For females with ID this pattern was reversed: chronically ill females with ID less often had 2 or more comorbidities than those without ID.

When focusing on the characteristics of these comorbidities, comorbid diseases in the circulatory disease cluster (ICPC-code K) were most common in people with ID (not shown in figure; see Supplementary Table S4), disregarding of a diagnosis of either IHD, CVD, DM, or COPD. The most common comorbidity in all chronic diseases was hypertension, although occurrence was lower in people with than without ID. DM was also a common comorbidity in IHD, CVD, and COPD.

## Discussion

### Summary

This retrospective cross-sectional study examined chronic disease and comorbidity patterns in chronically ill patients with ID compared with those without ID in a Dutch general practice population in 2018. We found that although at group level chronic diseases appeared to be less prevalent in people with ID, considering age and sex revealed different patterns emerged and prevalence rates increased towards higher prevalence in those with ID. Adjusted PRs ranged from 0.74 (IHD) to 1.62 (DM). To illustrate, it seemed that already at age 18, patients with ID more often developed chronic diseases with or without comorbidities than those without ID. Although sex effects were less straightforward than age effects, results suggested that males with ID were most vulnerable: they most often had a diagnosis of chronic disease, and on top of that, they also more often had multiple comorbidities. For females, the opposite pattern emerged: the least often they were diagnosed with a chronic disease or had 2 or more comorbidities than females without ID.

### Strengths and limitations

This study is the first to provide a large-scale comparative insight into comorbidity patterns in chronically ill people with and without ID in Dutch general practice. By linking a primary care database with population data, we were able to identify more individuals with ID than would be possible through their GP records alone, thereby overcoming challenges in recognizing people with ID in population datasets .^28^ Because of the large scale and linking possibilities, it is likely that our combined dataset provides reliable insight into clinical practice and the health of people with ID obtaining care in general practice.

Although data linkage allowed to identify more individuals with ID, the available data did not contain information on ID aetiology. This prevented us from differentiating between syndromes or ID severity, despite signs that ID severity is related to multimorbidity and specific syndromes have increased risk of specific chronic diseases.^13, 21^ Future research could therefore provide a more in-depth insight by taking into account ID aetiology.

Next, this study utilized documented diagnoses to assess disease prevalence. Although these are deemed reliable,^29^ under-recognition of health conditions in people with ID remains a widespread concern.^30^ Chronic diseases and comorbidities in people with ID may therefore not always be recognized, implying actual prevalence rates may even be higher. One way of gaining more thorough insights is to supplement data with screenings by health professionals,^31^ such as health assessment instruments specifically developed for people with ID to assist in diagnosing health conditions (e.g. PROSPER-ID ^31^).

### Comparison with existing literature

CVD, DM, and COPD were 1.5 times more prevalent in people with than without ID. These diseases, as well as IHD, are all lifestyle related.^32^ People with ID have unhealthy lifestyles more often than those without ID,^33^ which could possibly explain the higher DM and COPD prevalence rates. However, it does not explain the lower IHD prevalence in those with ID. It could be that cardiovascular diseases are more often managed or seen in secondary (hospital) or tertiary care settings, rather than in primary care. Some studies indeed reported higher hospitalization for cardiovascular disease prevalence in people with ID.^34, 35^

At group level chronic disease prevalence appeared to be lower in people with ID. Solely when considering age and sex, chronic disease prevalence was higher in people with than those without ID. Demographic differences between the two groups may thus influence chronic disease prevalence. How age and sex precisely affect chronic disease patterns should be further explored, though it can be the case that chronic diseases may develop at younger age in people with ID, under influence of factors such as genetics, early frailty, medicine use, or lifestyle .^5, 30, 33, 36^

We confirmed previous findings on the importance of age in the prevalence of chronic diseases, such as the pattern of older people with ID more often having a chronic disease,^8, 30^ even at younger age. This indicates that frailty occurs earlier than in the general population^6^: at younger age, chronic diseases as well as comorbidities were more prevalent in people with ID than in the general population. At age 50–64 years, frailty occurred in similar rates in people with ID than in those without ID aged 65 years or older.^6^ However, the high occurrence of COPD at young age (18–24 years) may also be due to wrongly coding asthma as COPD as it can be difficult to make the distinction between both in people with ID.^37^

Having two or more comorbidities is relatively common in chronically ill people with ID,^11, 13-15, 18, 19^ even more so at younger age.^12^ However, as most studies focus solely on older adults,^13, 15, 18^ comparison is difficult. This high prevalence could be associated with the congenital or genetic aetiology of the ID (i.e., epilepsy in people with ID or hypothyroidism in people with Down syndrome).^36, 38, 39^

Although our results seem to suggest that people with ID from age 65 onwards are more healthy than those without ID, a healthy survivor effect may have occurred. This means that although life expectancy of people with ID has increased, it is still lower than that of the general population.^5^ The ID-population aged 65 years and older in our dataset may therefore comprise a relatively more healthy group.

While this study reported important findings regarding sex differences between those with and without ID in the prevalence of chronic diseases and comorbidities, literature is scarce. Our finding that males with ID most often had a diagnosis of a chronic disease can therefore not easily be compared to existing literature. Previous research is inconclusive on chronic conditions being more prevalent in males or females: it appears to depend on the type of conditions studied.^15, 17, 19^ Additionally, unlike the current study, having two or more health conditions was found to be more common in females with ID,^12, 17, 20^ or others did not find a sex effect.^13, 16^ Literature is thus inconclusive on sex effects in chronic diseases, however, this study highlights the importance of considering sex differences in chronic diseases between people with and without ID.

Characteristics of comorbidities in chronically ill people with ID are less often studied. We found that most comorbidities were of the circulatory system. Similar to our findings, studies reported lower prevalence of comorbidities in cardiovascular clusters in people with ID.^15, 17^ As we confirmed previous literature on hypertension being a highly prevalent comorbidity in chronic diseases in people with ID,^15, 17^ the lower prevalence of cardiovascular comorbidities may be due to underdiagnosis.

### Implications for clinical practice

People with ID display different disease patterns than the usual patients seen in general practice. Younger people with ID are particularly burdened: they more often have more chronic diseases and more comorbidities. These findings therefore aid general practitioners to develop greater awareness of differences between people with and without ID. This awareness is essential and underlying in providing suitable and tailored chronic disease care for people with ID.^21, 22^ By increased collaboration between general practitioners and care professionals in specialized ID-care, recognizing and treating chronic diseases and comorbidities within ID-patients can be optimized.^13^

In addition, suitable chronic disease prevention and treatment could relieve the high burden of comorbidities in people with ID as presented in this study. It is therefore essential to create awareness on health behaviours and engage people with ID in lifestyle alterations to decrease body weight.^33, 40^ When interventions are combined with (structural) proactive risk assessments, diseases can be diagnosed at earlier stage .^31^

## Conclusions

Patterns of chronic diseases with and without comorbidities were different in adults with ID compared with those without ID. It seems that people with ID developed chronic diseases with or without comorbidities at a younger age than people without ID. Males with ID carried the highest burden: they most often had a chronic disease as well as multiple comorbidities. Due to these important differences with the general population, general practitioners should be aware of chronic diseases and comorbidities especially in younger people with ID and males with ID. Pro-active health assessments can therefore be used to timely recognise health conditions. This could be an important addition to regular chronic disease guidelines to acknowledge the earlier onset of chronic diseases and comorbidities in people with ID, and ensure their equal chances to high-quality care. This way, person-centred care can be provided, ultimately the basis for reducing existing health inequities.

## Supplementary Material

cmac042_suppl_Supplementary_Material

cmac042_suppl_Supplementary_Checklist

## Data Availability

The data underlying this article were provided by Nivel by permission. Data will be shared on request to the corresponding author with permission of Nivel.
