# Supplementary material for: Chronic diseases and comorbidities in adults with and without intellectual disabilities: comparative cross-sectional study in Dutch general practice
Source: Fam Pract. 2022 May 17;39(6):1056–62. doi: 10.1093/fampra/cmac042 (PMC9680667; doi:10.1093/fampra/cmac042)
Supplement: cmac042_suppl_Supplementary_Material [file cmac042_suppl_supplementary_material.docx]

**Supplementary files**

Table S1. Selection of four chronic diseases and accompanying ICPC codes

| Chronic disease | ICPC code |
| --- | --- |
| Ischaemic heart disease |  |
| Angina pectoris | K74 |
| Myocardial infarction | K75 |
| Other/chronic ischemic heart disease | K76 |
| Cerebrovascular disease |  |
| Transient ischemic attack | K89 |
| Cerebrovascular accident | K90 |
| Diabetes mellitus | T90 |
| Chronic obstructive pulmonary disease |  |
| Chronic bronchitis/bronchiectasis | R91 |
| Emphysema/COPD | R95 |

Table S2. Selection of 108 chronic comorbidities and accompanying ICPC codes

| ICPC code | Comorbidity | ICPC code | Comorbidity |
| --- | --- | --- | --- |
| A28 | Limited function/disability NOS | N28 | Limited function/disability neurological |
| A79 | Malignancy not otherwise specified | N70 | Poliomyelitis |
| A90 | Congenital anomaly otherwise specified/multiple | N74 | Malignant neoplasm nervous system |
| B28 | Limited function/disability blood/lymphatic system | N85 | Congenital anomaly neurological |
| B72 | Hodgkin disease/lymphoma | N86 | Multiple sclerosis |
| B73 | Leukaemia | N87 | Parkinsonism |
| B74 | Malignant neoplasm blood other | N88 | Epilepsy |
| B78 | Hereditary haemolytic anaemia | P28 | Limited function/disability psychological |
| B79 | Congenital anomaly blood/lymphatic system other | P70 | Dementia |
| B83 | Purpura/coagulation defect | P72 | Schizophrenia |
| B90 | HIV-infection/aids | P80 | Personality disorder |
| D28 | Limited function/disability digestive | R28 | Limited function/disability respiratory system |
| D74 | Malignant neoplasm stomach | R84 | Malignant neoplasm bronchus/lung |
| D75 | Malignant neoplasm colon/rectum | R85 | Malignant neoplasm respiratory, other |
| D76 | Malignant neoplasm pancreas | R89 | Congenital anomaly respiratory tract |
| D77 | Malignant neoplasm digestive system other/not otherwise specified | R91 | Chronic bronchitis/bronchiectasis |
| D81 | Congenital anomaly digestive system | R95 | Chronic obstructive pulmonary disease |
| D92 | Diverticular disease | R96 | Asthma |
| D94 | Chronic enteritis/ulcerative colitis | S28 | Limited function/disability skin |
| D97 | Liver disease not otherwise specified | S77 | Malignant neoplasm of skin |
| F28 | Limited function/disability eye | S81 | Haemangioma/lymphangioma |
| F81 | Congenital abnormality eye/other | S83 | Congenital skin anomaly other |
| F83 | Retinopathy | S87 | Dermatitis/atopic eczema |
| F84 | Macular degeneration | S91 | Psoriasis |
| F91 | Refractive error | T28 | Limited function/disability endocrine system/metabolism/nutrition |
| F93 | Glaucoma | T71 | Malignant neoplasm thyroid |
| F94 | Blindness | T78 | Thyroglossal duct/cyst |
| H28 | Limited function/disability ear | T80 | Congenital anomaly endocrine/ metabolic system |
| H80 | Congenital anomaly of ear | T81 | Goitre |
| H83 | Otosclerosis | T86 | Hypothyroidism/myxoedema |
| H84 | Presbycusis | T90 | Diabetes mellitus |
| H85 | Acoustic trauma | T92 | Gout |
| H86 | Deafness | T93 | Lipid disorder |
| K28 | Limited function/disability cardiovascular | U28 | Limited function/disability urinary tracts |
| K73 | Congenital anomaly cardiovascular system | U75 | Malignant neoplasm of kidney |
| K74 | Ischaemic heart disease with angina pectoris | U76 | Malignant neoplasm of bladder |
| K76 | Ischaemic heart disease without angina pectoris | U77 | Malignant neoplasm urinary other |
| K77 | Heart failure | U85 | Congenital anomaly urinary tracts |
| K82 | Pulmonary heart disease | U88 | Glomerulonephritis/nephrosis |
| K86 | Hypertension uncomplicated | W28 | Limited function/disability as a result of pregnancy |
| K87 | Hypertension complicated | W72 | Malignant neoplasm related to pregnancy |
| K90 | Stroke/cerebrovascular accident | W76 | Congenital anomaly complicating pregnancy |
| K91 | Atherosclerosis/PVD | X28 | Limited function/disability female genitals |
| K92 | Pulmonary embolism | X75 | Malignant neoplasm cervix |
| L28 | Limited function/disability musculoskeletal | X76 | Malignant neoplasm breast female |
| L82 | Congenital anomaly musculoskeletal system | X77 | Malignant neoplasm genital other (f) |
| L84 | Back syndrome without radiating pain | X83 | Congenital anomaly genital female |
| L85 | Acquired deformity of spine | X88 | Fibrocystic disease breast |
| L88 | Rheumatoid/seropositive arthritis | Y28 | Limited function/disability male genitals |
| L89 | Osteoarthrosis of hip | Y77 | Malignant neoplasm of prostate |
| L90 | Osteoarthrosis of knee | Y78 | Malignant neoplasm male genital other |
| L91 | Osteoarthrosis other | Y82 | Hypospadias |
| L95 | Osteoporosis | Y84 | Congenital genital anomaly other (m) |
| L98 | Acquired deformity of limb | Z28 | Limited function/disability social problems |

Table S3. Prevalence of chronic diseases for people with and without intellectual disabilities by sex and 5-year age groups in N (%)

|  | IHD | | CVD | | DM | | COPD | |
| --- | --- | --- | --- | --- | --- | --- | --- | --- |
|  | ID  N=18,114 | No ID  N=1,093,995 | ID  N=18,114 | No ID  N=1,093,995 | ID  N=18,114 | No ID  N=1,093,995 | ID  N=18,114 | No ID  N=1,093,995 |
| Unadjusted PR (95% CIs) | **0.466****  **(0.425; 0.510)** | | **0.692****  **(0.630; 0.760)** | | **1.076****  **(1.026; 1.128)** | | 0.975  (0.907; 1.047) | |
| Adjusted PR (95% CIs) | **0.744****  **(0.681; 0.812)** | | **1.118***  **(1.020; 1.227)** | | **1.616****  **(1.544; 1.692)** | | **1.517****  **(1.415; 1.626)** | |
| Total group | 461 (2.5) | 59,808 (5.5) | 431 (2.4) | 37,613 (3.4) | 1,584 (8.7) | 88,925 (8.1) | 736 (4.1) | 45,611 (4.2) |
| Sex |  |  |  |  |  |  |  |  |
| Males | 325 (3.1) | 37,407 (7.0) | 255 (2.5) | 18,978 (3.6) | 874 (8.5) | 47,125 (8.8) | 428 (4.1) | 22,713 (4.3) |
| Females | 136 (1.7) | 22,401 (4.0) | 176 (2.3) | 18,635 (3.3) | 710 (9.1) | 41,800 (7.5) | 308 (4.0) | 22,898 (4.1) |
| Age groups |  |  |  |  |  |  |  |  |
| 18-24 years | <10 | 26 (<0.1) | 14 (3.2) | 84 (0.2) | 38 (2.4) | 694 (0.8) | 24 (3.3) | 383 (0.8) |
| 25-29 years | <10 | 39 (0.1) | 14 (3.2) | 91 (0.2) | 58 (3.7) | 588 (0.7) | 30 (4.1) | 344 (0.8) |
| 30-34 years | <10 | 65 (0.1) | 12 (2.8) | 149 (0.4) | 44 (2.8) | 786 (0.9) | 21 (2.9) | 361 (0.8) |
| 35-39 years | <10 | 181 (0.3) | 13 (3.0) | 283 (0.8) | 75 (4.7) | 1176 (1.3) | 19 (2.6) | 472 (1.0) |
| 40-44 years | 15 (3.3) | 494 (0.8) | 14 (3.2) | 464 (1.2) | 107 (6.8) | 2024 (2.3) | 37 (5.0) | 756 (1.7) |
| 45-49 years | 31 (6.7) | 1406 (2.4) | 33 (7.7) | 1129 (3.0) | 161 (10.2) | 4023 (4.5) | 53 (7.2) | 1661 (3.6) |
| 50-54 years | 64 (13.9) | 2965 (5.0) | 56 (13.0) | 1895 (5.0) | 224 (14.1) | 6336 (7.1) | 102 (13.9) | 2817 (6.2) |
| 55-59 years | 93 (20.2) | 4984 (8.3) | 71 (16.5) | 2835 (7.5) | 278 (17.6) | 8847 (9.9) | 143 (19.4) | 4502 (9.9) |
| 60-64 years | 85 (18.4) | 7050 (11.8) | 86 (20.0) | 3727 (9.9) | 235 (14.8) | 11025 (12.4) | 117 (15.9) | 6235 (13.7) |
| 65-69 years | 82 (17.8) | 8708 (14.6) | 51 (11.8) | 4827 (12.8) | 182 (11.5) | 12803 (14.4) | 94 (12.8) | 6961 (15.3) |
| 70-74 years | 44 (9.5) | 10184 (17.0) | 25 (5.8) | 5984 (15.9) | 108 (6.8) | 14113 (15.9) | 59 (8.0) | 7268 (15.9) |
| 75-79 years | 19 (4.1) | 8827 (14.8) | 24 (5.6) | 5515 (14.7) | 50 (3.2) | 11058 (12.4) | 21 (2.9) | 5635 (12.4) |
| 80+ years | 14 (3.0) | 14879 (24.9) | 18 (4.2) | 10630 (28.3) | 24 (1.5) | 15452 (17.4) | 16 (2.2) | 8216 (18.0) |

Table S4. Amount of chronically ill people with and without intellectual disabilities having 2 or more comorbidities and comorbidity characteristics by sex and 5-year age groups in N (%)

|  | IHD | | CVD | | DM | | COPD | |
| --- | --- | --- | --- | --- | --- | --- | --- | --- |
|  | ID | No ID | ID | No ID | ID | No ID | ID | No ID |
| People having 2+ comorbidities |  |  |  |  |  |  |  |  |
| Total group | 395 (85.7) | 48645 (81.3) | 363 (84.2) | 30865 (82.1) | 1237 (78.1) | 66995 (75.3) | 560 (76.1) | 34688 (76.1) |
| Sex |  |  |  |  |  |  |  |  |
| Males | 270 (68.4) | 29021 (59.7) | 204 (56.2) | 15118 (49.0) | 645 (52.1) | 33872 (50.6) | 317 (56.6) | 16808 (48.5) |
| Females | 125 (31.6) | 19624 (40.3) | 159 (43.8) | 15747 (51.0) | 592 (47.9) | 33123 (49.4) | 243 (43.4) | 17880 (51.5) |
| Age groups |  |  |  |  |  |  |  |  |
| 18-24 years | <10 | 10 (<0.1) | <10 | 26 (0.1) | 21 (1.7) | 101 (0.2) | 14 (2.5) | 89 (0.3) |
| 25-29 years | <10 | 13 (<0.1) | <10 | 22 (0.1) | 35 (2.8) | 116 (0.2) | 15 (2.7) | 87 (0.3) |
| 30-34 years | <10 | 24 (<0.1) | <10 | 48 (0.2) | 22 (1.8) | 180 (0.3) | 10 (1.8) | 105 (0.3) |
| 35-39 years | <10 | 57 (0.1) | <10 | 115 (0.4) | 53 (4.3) | 343 (0.5) | 13 (2.3) | 152 (0.4) |
| 40-44 years | 10 (2.5) | 214 (0.4) | <10 | 211 (0.7) | 65 (5.3) | 775 (1.2) | 26 (4.6) | 278 (0.8) |
| 45-49 years | 21 (5.3) | 693 (1.4) | 29 (8.0) | 562 (1.8) | 113 (9.1) | 1844 (2.8) | 31 (5.5) | 735 (2.1) |
| 50-54 years | 47 (11.9) | 1696 (3.5) | 47 (12.9) | 1135 (3.7) | 180 (14.6) | 3493 (5.2) | 72 (12.9) | 1477 (4.3) |
| 55-59 years | 79 (20.0) | 3267 (6.7) | 59 (16.3) | 1884 (6.1) | 227 (18.4) | 5728 (8.5) | 115 (20.5) | 2798 (8.1) |
| 60-64 years | 78 (19.7) | 5183 (10.7) | 76 (20.9) | 2790 (9.0) | 199 (16.1) | 8080 (12.1) | 97 (17.3) | 4406 (12.7) |
| 65-69 years | 75 (19.0) | 6921 (14.2) | 50 (13.8) | 3940 (12.8) | 158 (12.8) | 10199 (15.2) | 82 (11.1) | 5552 (12.2) |
| 70-74 years | 43 (10.9) | 8632 (17.7) | 23 (6.3) | 5190 (16.8) | 96 (7.8) | 11942 (17.8) | 49 (6.7) | 6182 (13.6) |
| 75-79 years | 18 (4.6) | 7891 (16.2) | 23 (6.3) | 4986 (16.2) | 45 (3.6) | 9830 (14.7) | 21 (2.9) | 5074 (11.1) |
| 80+ years | 14 (3.5) | 14044 (28.9) | 15 (4.1) | 9956 (32.3) | 23 (1.9) | 14364 (21.4) | 15 (2.0) | 7753 (17.0) |
| Comorbidity characteristics |  |  |  |  |  |  |  |  |
| Circulatory diseases | 279 (60.5) | 39,484 (66.0) | 247 (57.3) | 26,431 (70.3) | 812 (51.3) | 60,645 (68.2) | 350 (47.6) | 27,558 (60.4) |
| Hypertension | 170 (36.9) | 25,028 (41.8) | 142 (32.9) | 16,563 (44.0) | 568 (35.9) | 44,147 (49.6) | 211 (28.7) | 17,529 (38.4) |
| DM | 165 (35.8) | 16,611 (27.8) | 102 (23.7) | 8,861 (23.6) |  |  | 166 (22.6) | 9,137 (20.0) |
